# Single-cell and transcriptomic profiling reveal stemness-driven immune evasion in obstructive sleep apnea (OSA) associated lung cancer

**DOI:** 10.7150/jca.126708

**Published:** 2026-01-01

**Authors:** Yu-Wei Liu, Chi-Jen Wu, Kai-Fu Chang, Yung-Kuo Lee, Hui-Ru Lin, Ching-Chung Ko, Chung-Bao Hsieh, Chih-Hsuan Chang, Chung-Hsien Lin, Bo-Sheng Chen, Dahlak Daniel Solomon, Sachin Kumar, Neethu Palekkode, Ayman Fathima, Do Thi Minh Xuan, Ngoc Uyen Nhi Nguyen, Junanda Waikhom, Chien-Han Yuan, Yuen-Jung Wu

**Affiliations:** 1 Division of Thoracic Surgery, Department of Surgery, Kaohsiung Medical University Hospital, Kaohsiung Medical University, Kaohsiung 80756, Taiwan.; 2 Nursing Department, Kaohsiung Armed Forces General Hospital, National Defense Medical University, Kaohsiung 80284, Taiwan.; 3 College of Nursing, Kaohsiung Medical University, Kaohsiung, 80708, Taiwan.; 4 Medical Laboratory, Medical Education and Research Center, Kaohsiung Armed Forces General Hospital, National Defense Medical University, Kaohsiung 80284, Taiwan.; 5 Division of Experimental Surgery Center, Department of Surgery, Tri-Service General Hospital, National Defense Medical University, Taipei, Taiwan.; 6 Institute of Medical Science and Technology, National Sun Yat-Sen University, Kaohsiung 80424, Taiwan.; 7 Department of Medical Imaging, Chi-Mei Medical Center, Tainan 710402, Taiwan.; 8 Department of Health and Nutrition, Chia Nan University of Pharmacy and Science, Tainan 71710, Taiwan.; 9 School of Medicine, College of Medicine, National Sun Yat-Sen University, Kaohsiung 80424, Taiwan.; 10 PhD Program for Cancer Molecular Biology and Drug Discovery, College of Medical Science and Technology, Taipei Medical University, Taipei 11031, Taiwan.; 11 Graduate Institute of Cancer Biology and Drug Discovery, College of Medical Science and Technology, Taipei Medical University, Taipei 11031, Taiwan.; 12 PhD Program for Cancer Molecular Biology and Drug Discovery, College of Medical Science and Technology, Taipei Medical University, Taipei 11031, Taiwan.; 13 Faculty of Applied Sciences and Biotechnology, Shoolini University of Biotechnology and Management Sciences, Himachal Pradesh, 173229, India.; 14 Department of Biotechnology, Mother Teresa Women's University, Kodaikanal, Tamil Nadu, 624101, India.; 15 Computer Engineering with specialization in Artificial Intelligence and Machine Learning, Presidency University, Yelahanka, Bengaluru 560064 India.; 16 Faculty of Pharmacy, Van Lang University, 69/68 Dang Thuy Tram Street, Ward 13, Binh Thanh District, Ho Chi Minh City 70000, Vietnam.; 17 Center for Regenerative Medicine, University of South Florida Health Heart Institute, Tampa, Florida 33602, USA.; 18 Division of Cardiology, Department of Internal Medicine, Morsani School of Medicine, University of South Florida, Tampa, Florida 33602, USA.; 19 Department of Otolaryngology, Kaohsiung Armed Forces General Hospital, National Defense Medical University, Kaohsiung 80284, Taiwan.; 20 Department of Otolaryngology, National Defense Medical University, Taipei 11490, Taiwan.; 21 Department of Surgery, Kaohsiung Armed Forces General Hospital, National Defense Medical University, Kaohsiung 80284, Taiwan.

**Keywords:** obstructive sleep apnea, intermittent hypoxia, lung cancer, stemness, immune evasion, prognostic model, immunotherapy

## Abstract

Obstructive sleep apnea (OSA) is characterized by recurrent intermittent hypoxia (IH) and has been increasingly associated with lung cancer incidence and mortality. However, how IH-related biological programs relate to immune remodeling, stemness-associated phenotypes, and therapeutic resistance in lung cancer remains incompletely understood. We integrated single-cell RNA sequencing data from IH-exposed murine lung tissues (GSE301350) with bulk transcriptomic datasets from TCGA-LUAD and GSE31210 to examine hypoxia-associated cellular and transcriptional patterns. Stemness was quantified using CytoTRACE and transcriptome-based stemness scoring, and its associations with immune infiltration, immune checkpoint expression, TIDE scores, predicted drug sensitivity, and immunotherapy response were evaluated. A stemness-based prognostic model was constructed using LASSO Cox regression and validated in independent cohorts. Single-cell analysis revealed marked immune remodeling under intermittent hypoxia (IH), including expansion of effector T cells, and monocytes/macrophages, populations alongside reduced B cells and dendritic cells. In human LUAD cohorts, stemness-high tumors were associated with mitochondrial and metabolic stress-related transcriptional programs, and increased expression of immune checkpoint genes (PD-1, PD-L1, CTLA4, LAG3). Elevated stemness scores correlated with higher TIDE scores, poorer overall survival, and reduced predicted responsiveness to immunotherapy. LASSO modeling identified a six-gene stemness signature (EIF5A, MELTF, SEMA3C, CPS1, TCN1, SELENOK), that consistently stratified patients into high- and low-risk groups across TCGA and GSE31210 cohorts. Multivariate Cox regression confirmed the risk score as an independent prognostic factor. Drug sensitivity analyses further suggested that stemness-high tumors may exhibit increased susceptibility to selected kinase inhibitors (Dasatinib, A-770041) and metabolic modulators (Phenformin, Salubrinal). OSA-associated IH is linked to stemness-associated transcriptional plasticity, immune suppression, and adverse clinical outcomes in lung cancer. The identified stemness-based gene signature provides a robust prognostic biomarker and highlights potential therapeutic vulnerabilities, supporting integrative strategies that combine stemness and immune -targeted approaches with immunotherapy in OSA-associated lung cancer.

## 1. Introduction

Obstructive sleep apnea (OSA) is a highly prevalent sleep-related breathing disorder characterized by recurrent episodes of upper airway collapse and intermittent hypoxia (IH) 1-3. Epidemiological studies have established OSA as a risk factor for a broad spectrum of cardiometabolic diseases, including hypertension, type 2 diabetes, and atherosclerosis, as well as respiratory conditions such as chronic obstructive pulmonary disease and pulmonary hypertension 4-6. More recently, increasing attention has been directed toward the potential link between OSA and cancer. Clinical cohort studies and meta-analyses suggest that OSA severity, particularly nocturnal hypoxemia, correlates with higher incidence and mortality of lung cancer, yet the underlying molecular mechanisms remain poorly understood 7-10. Lung cancer remains the leading cause of cancer-related deaths worldwide, with non-small cell lung cancer (NSCLC) accounting for approximately 85% of all cases 11, 12. Despite advances in immunotherapy and targeted therapy, the prognosis of advanced NSCLC remains unsatisfactory, highlighting the urgent need for improved biomarkers and mechanistic insights 13, 14. Hypoxia is a well-recognized hallmark of the tumor microenvironment (TME), promoting epithelial-to-mesenchymal transition, angiogenesis, immune suppression, and therapeutic resistance 15, 16. Given that OSA subjects experience recurrent systemic and tissue-level hypoxic stress, intermittent hypoxia may act as a “priming factor” that accelerates lung tumorigenesis and shapes the TME toward malignancy 10, 17. However, most prior studies have focused on bulk transcriptomic or animal models, leaving the cell-type-specific effects of IH largely unexplored 18, 19.

Recent advances in single-cell RNA sequencing (scRNA-seq) provide unprecedented opportunities to dissect the heterogeneity of lung tissues under OSA-associated IH conditions 20, 21. By resolving cell-type-specific transcriptional programs, scRNA-seq allows the identification of altered immune infiltration, stemness-associated reprogramming, and immunometabolic shifts that bulk analyses cannot capture 22. In particular, cellular stemness, the degree to which tumor and immune cells adopt progenitor-like transcriptional states has emerged as a critical determinant of tumor progression, immune evasion, and therapy response. Computational approaches such as CytoTRACE and integrative stemness scoring now enable quantification of stemness at both single-cell and bulk-tumor levels, facilitating the construction of prognostic models with direct clinical applicability 23-25. Candidate genes and pathways highlighted in this study were prioritized using a quantitative, multi-criteria framework, incorporating stemness association, statistical robustness, cross-dataset consistency, and relevance to hypoxia and immune regulation, rather than isolated statistical significance.

**Figure 1:**
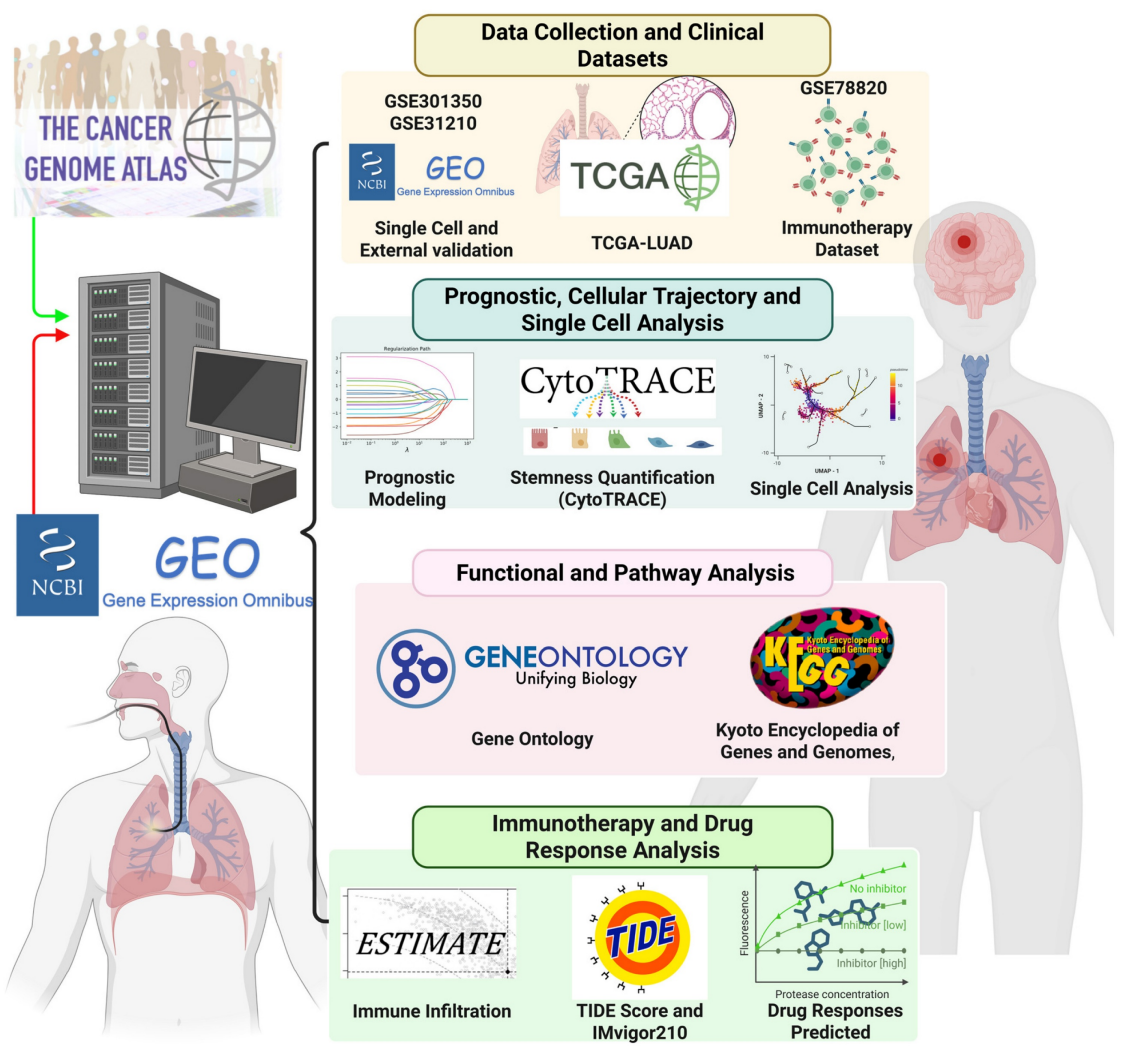
** Study workflow and integrative analyses for OSA-associated lung cancer.** Study workflow and integrative analyses for OSA-associated lung cancer. Overview of the computational and experimental strategy. Multi-omics data (TCGA-LUAD, GSE301350, GSE31210, GSE78820) and single-cell RNA-seq datasets were integrated to quantify stemness (CytoTRACE, stemness scores), characterize immune infiltration (CIBERSORT, ESTIMATE), and assess functional pathways (GO ontology, and KEGG,). Prognostic modeling was performed using LASSO Cox regression and validated in independent cohorts. Immunotherapy response was evaluated using TIDE and IMvigor210 datasets, and drug sensitivity was predicted using GDSC data. The pipeline highlights the integration of stemness quantification, immune landscape profiling, and therapeutic prediction in OSA-associated lung cancer.

In this study, we integrated scRNA-seq data from OSA-associated IH lung models (GSE301350) with bulk transcriptomic datasets (TCGA-LUAD and external validation cohorts) to investigate the role of stemness in OSA-related lung cancer. Through comprehensive analyses, including immune infiltration deconvolution, checkpoint gene profiling, TIDE assessment, and drug sensitivity prediction, we aimed to delineate the molecular mechanisms linking OSA-driven hypoxia to tumor progression and therapeutic resistance. Furthermore, we developed and validated a stemness-based prognostic model using LASSO Cox regression, demonstrating its independent predictive value and translational potential in guiding personalized therapy (Figure 1) 26, 27. Our work provides novel insights into how OSA-associated intermittent hypoxia may reprograms the lung microenvironment through stemness-associated and immune-related mechanisms, thereby enhancing cancer susceptibility and progression. These findings not only improve mechanistic understanding of the OSA-cancer link but also identify actionable biomarkers and therapeutic vulnerabilities that could inform future precision oncology strategies for patients with OSA-related lung cancer. We emphasize that this study does not assume direct molecular equivalence between murine intermittent hypoxia models and human lung adenocarcinoma. Instead, the murine single-cell RNA-seq data were used as a mechanistic discovery framework to characterize hypoxia-responsive cellular states and immune remodeling, while independent human LUAD transcriptomic cohorts were leveraged to assess the clinical relevance of stemness-associated transcriptional programs. This integrative strategy allows hypoxia-linked biological themes identified in experimental models to be contextualized and validated at the prognostic and immunological level in human disease, without implying direct cross-species gene-level concordance.

## 2. Methods

### 2.1 Data acquisition and preprocessing

The single-cell RNA-seq dataset GSE301350, comprising lung tissues from mice exposed to intermittent hypoxia (IH) or normoxia (Nx), was downloaded from the Gene Expression Omnibus (GEO). Raw count matrices were processed using the Seurat R package (v4.4.1). Cells with fewer than 200 detected genes, more than 5,000 detected genes, or > 10% mitochondrial gene content were excluded. Gene expression matrices were normalized and log-transformed, followed by principal component analysis (PCA) and batch effect correction using Harmony 28-30. Dimensionality reduction was performed using t-distributed stochastic neighbor embedding (t-SNE) and uniform manifold approximation and projection (UMAP).

### 2.2 Cell-type annotation and marker gene identification

Cell clustering was performed with the Seurat “FindClusters” function at a resolution of 0.5-1.2. Marker genes for each cluster were identified by the “FindMarkers” function using Wilcoxon rank-sum testing with thresholds of |log2 fold change| > 0.25 and adjusted p < 0.05. Cell types were annotated based on canonical lineage markers, cross-referenced with the CellMarker database and published literature 31-35.

### 2.3 Stemness estimation and functional enrichment

Cellular differentiation potential at the single-cell level was inferred using CytoTRACE, with higher scores indicating greater transcriptional similarity to progenitor-like states. At the bulk-transcriptomic level stemness scores for TCGA-LUAD and GSE31210 samples were calculated by summing the weighted expression of stemness-associated product of gene using pre-defined coefficients. Functional enrichment analyses were conducted to identify pathway-associated transcriptional programs linked to stemness and hypoxia-related phenotypes. Gene Ontology biological process (GO-BP) and KEGG pathway gene sets were analyzed using the *clusterProfiler* package (v4.10.0). Enrichment results were interpreted as coordinated gene expression patterns rather than direct evidence of biological pathway activation or mechanistic causality. Statistical significance was assessed using adjusted p-values, with pathways meeting an adjusted p-value < 0.05 and false discovery rate (FDR) < 0.25 considered significantly enriched 36-41.

### 2.4 Survival analysis and prognostic model construction

Differentially expressed genes between high- and low-stemness groups were identified using limma (v3.54.2). Genes associated with overall survival were first screened by univariate Cox regression (p < 0.05). To minimize overfitting, least absolute shrinkage and selection operator (LASSO) Cox regression was applied using the glmnet package with 10-fold cross-validation. A multigene prognostic signature was established, and a risk score was calculated as the sum of each gene's expression weighted by its regression coefficient. Patients were stratified into high- and low-risk groups using the median risk score. Model performance was evaluated using Kaplan-Meier survival analysis and time-dependent receiver operating characteristic (ROC) curves (timeROC package).

### 2.5 Independent validation

The prognostic model was validated in the GSE31210 dataset. Risk scores were computed using the same formula derived from TCGA-LUAD. Kaplan-Meier and ROC analyses were performed to assess predictive accuracy. Univariate and multivariate Cox regression analyses incorporating age, sex, TNM stage, and clinical stage were conducted to determine independent prognostic value 42-46.

### 2.6 Immune infiltration and checkpoint analysis

Immune cell proportions were estimated using CIBERSORT with 22 leukocyte subsets and 1,000 permutations. Stromal and immune scores were calculated using the ESTIMATE algorithm. Immune checkpoint expression was compared between stemness-defined groups using Wilcoxon rank-sum tests. Associations between stemness score and immune checkpoint expression were assessed using Pearson correlation.

### 2.7 TIDE and immunotherapy response prediction

The Tumor Immune Dysfunction and Exclusion (TIDE) algorithm was used to estimate tumor immune escape potential. Associations between TIDE and stemness scores were evaluated using Spearman correlation. External immunotherapy cohorts with clinical response data were used to assess whether stemness risk groups predicted treatment outcomes, with patients categorized as responders (complete response [CR] or partial response [PR]) or non-responders (stable disease [SD] or progressive disease [PD]) 47-51.

### 2.8 Drug sensitivity analysis

Drug response prediction was performed using the oncoPredict package. Estimated half-maximal inhibitory concentration (IC50) values for compounds in the GDSC database were compared between high- and low-risk groups using Wilcoxon testing. Candidate drugs were selected based on consistent differences across cohorts and biological relevance to stemness-related pathways 52-56.

### 2.9 Statistical analysis

All statistical analyses were performed in R (v4.4.1). Continuous variables were compared using the Wilcoxon rank-sum test or Student's t-test, as appropriate. Correlations were assessed by Pearson or Spearman coefficients. Survival differences were determined using the log-rank test. A two-tailed p-value < 0.05 was considered statistically significant unless otherwise specified 57-61.

## 3. Results

### 3.1 Differential gene expression and LASSO modeling identify stemness-associated prognostic signatures in OSA-related lung cancer

**Figure 2:**
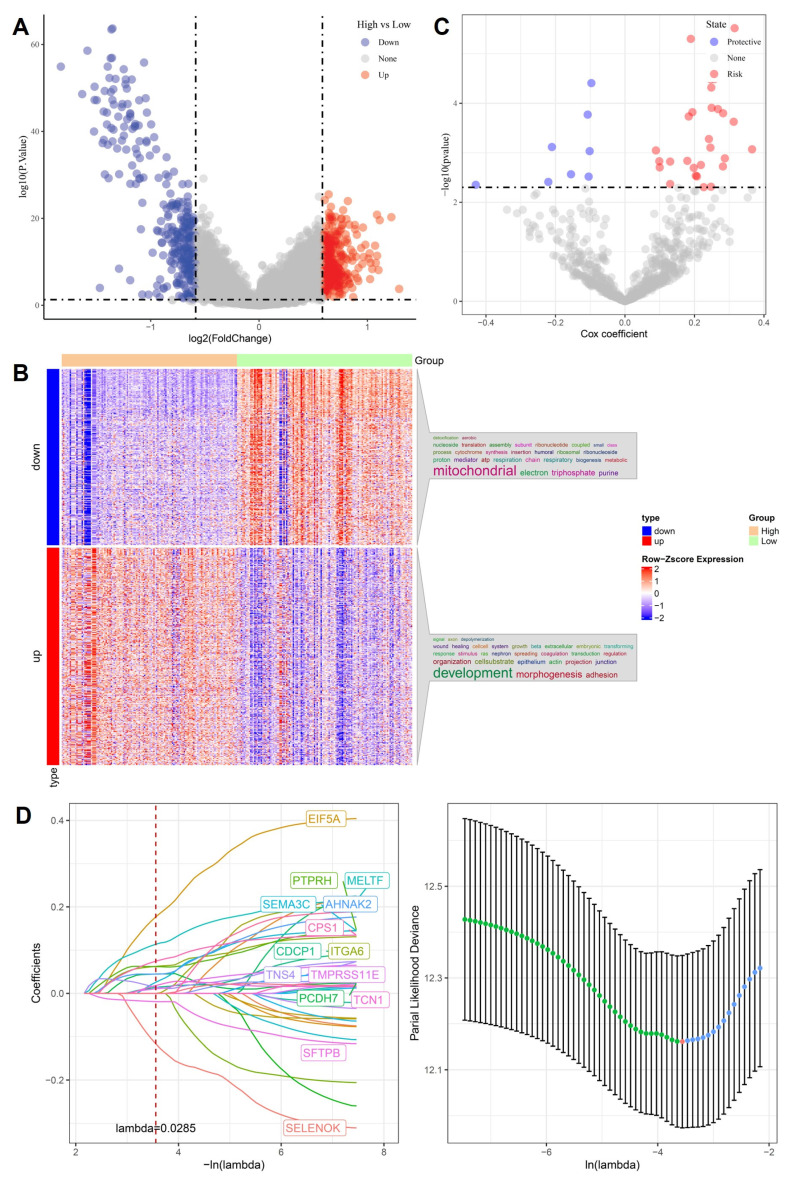
** Identification of stemness-associated prognostic genes.** (A) Volcano plot of differentially expressed genes (high vs. low stemness groups). (B) Heatmap of up- and downregulated genes, enriched in mitochondrial and developmental pathways. (C) Univariate Cox analysis highlighting risk and protective genes. (D) LASSO regression identifying optimal prognostic gene set with cross-validation curve.

To uncover molecular mediators of stemness-driven progression in OSA-related lung cancer, we first compared transcriptomes between high- and low-stemness tumors. Volcano plot analysis revealed hundreds of differentially expressed genes (DEGs), with significant upregulation of developmental regulators and suppression of mitochondrial/respiratory chain genes (Figure 2A). Heatmap clustering confirmed that stemness-high tumors were characterized by enhanced expression of adhesion, morphogenesis, and extracellular matrix remodeling genes, while low-stemness tumors were enriched in oxidative phosphorylation and electron transport pathways (Figure 2B). We next performed univariate Cox regression to assess the prognostic impact of DEGs. Several genes, including EIF5A and MELTF, emerged as high-risk factors, while others, such as SFTPB and SELENOK, displayed protective associations (Figure 2C). This dichotomy highlights the complex transcriptional landscape in which stemness-high tumors harness developmental pathways to enhance aggressiveness while silencing mitochondrial guardians that maintain cellular homeostasis. To refine these observations into a clinically actionable model, we applied LASSO Cox regression. Cross-validation identified a parsimonious panel of 15 genes with optimal prognostic value (Figure 2D). This stemness-associated signature integrates developmental drivers such as PTPRH, AHNAK2, PCDH7, metabolic regulators CPS1, SFTPB, and hypoxia-responsive factors such as TCN1, TMPRSS11E, reflecting the multifaceted nature of OSA-induced tumor evolution. Importantly, the predictive capacity of this model provides a robust framework for stratifying patients by risk, linking stemness biology to clinical outcomes. Collectively, these analyses establish a mechanistic bridge between OSA-related hypoxia, stemness reprogramming, and clinical prognosis. By pinpointing gene signatures tied to both mitochondrial dysfunction and developmental activation, the LASSO-derived model offers not only prognostic insights but also potential therapeutic targets to disrupt the stemness-hypoxia-immune axis in lung cancer.

### 3.2 Stemness-associated gene signature robustly predicts survival across training and validation cohorts

**Figure 3:**
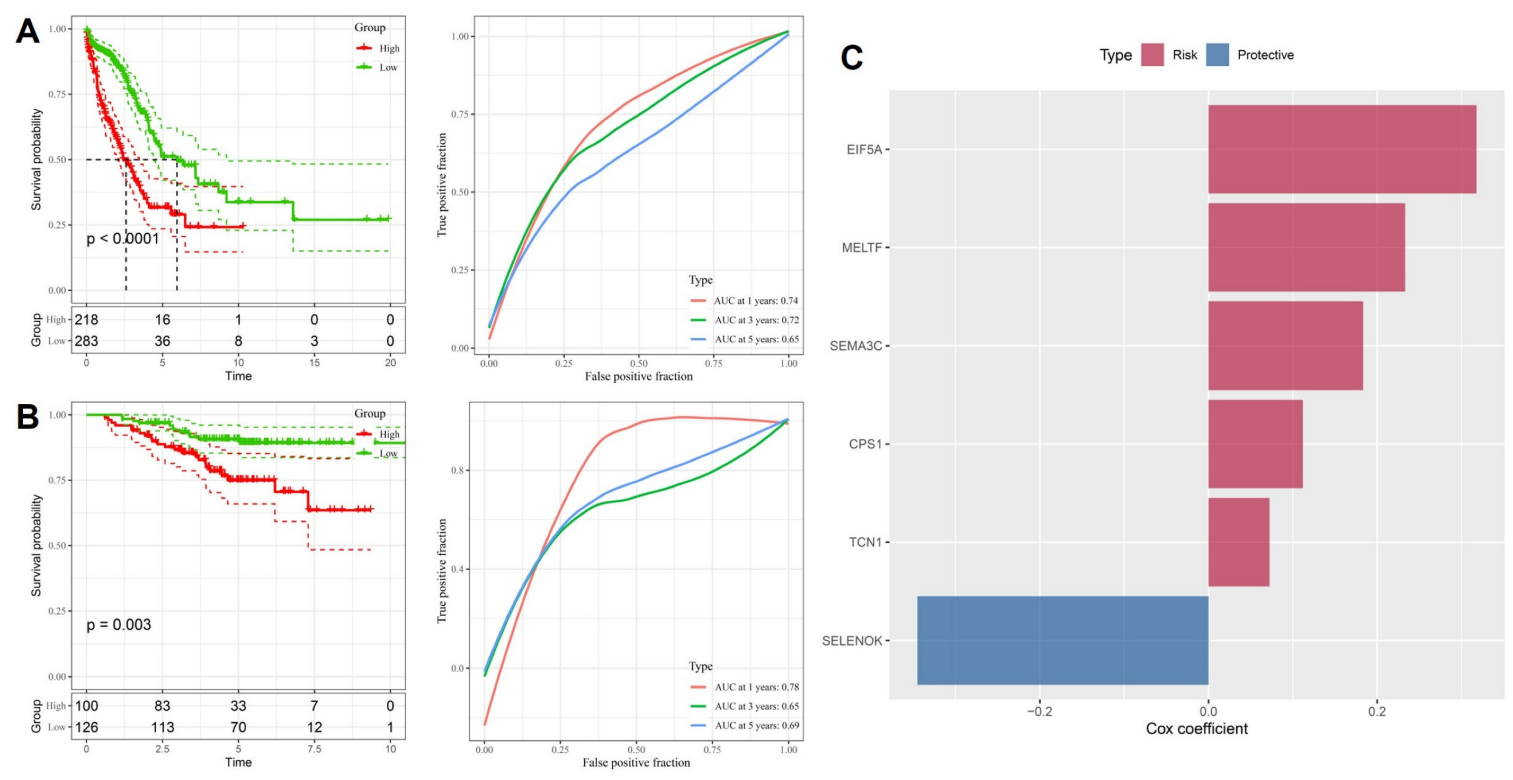
** Prognostic performance of the stemness-based risk model.** (A) Kaplan-Meier and ROC analysis in TCGA-LUAD training cohort, showing worse survival in high-risk patients and good predictive accuracy (AUC = 0.74, 0.72, 0.65 for 1-, 3-, and 5-year survival). (B) Validation in GSE31210 cohort confirmed survival discrimination and predictive performance (AUC = 0.78, 0.65, 0.69). (C) Cox coefficients of selected genes in the final model, with EIF5A, MELTF, SEMA3C, CPS1, and TCN1 as risk factors, and SELENOK as protective.

To validate the prognostic value of the stemness-associated signature derived from LASSO analysis, we first applied it to the TCGA-LUAD training dataset. Patients stratified into high- and low-risk groups demonstrated markedly divergent outcomes, with the high-risk group exhibiting significantly worse overall survival (p < 0.0001, Figure 3A). Time-dependent ROC analysis confirmed good predictive performance, with AUC values of 0.74, 0.72, and 0.65 for 1-, 3-, and 5-year survival, respectively, indicating that the model retains both short- and medium-term prognostic utility. Independent validation in the GSE31210 cohort yielded consistent results, with high-risk patients again showing significantly poorer survival (p = 0.003, Figure 3B). Predictive accuracy was comparable, with AUC values of 0.78, 0.65, and 0.69 across 1-, 3-, and 5-year timepoints, demonstrating the robustness and generalizability of the model across different patient populations. Examination of gene-specific Cox coefficients highlighted both risk and protective components of the model (Figure 3C). EIF5A, MELTF, SEMA3C, CPS1, and TCN1 were identified as risk-promoting genes, each associated with adverse prognosis and consistent with their roles in proliferation, metabolic rewiring, and hypoxia responses. In contrast, SELENOK emerged as a protective factor, potentially linked to its role in antioxidant defense and maintenance of cellular homeostasis. Together, these results confirm that the stemness-driven gene signature derived from OSA-related hypoxia biology provides a reliable and externally validated prognostic model. By integrating developmental regulators, metabolic genes, and hypoxia-responsive factors, the model captures the multifaceted influence of intermittent hypoxia on lung tumor evolution. Importantly, the reproducibility of its predictive power across TCGA and GSE31210 underscores its translational potential as a clinical tool for patient risk stratification and treatment planning.

### 3.3 Stemness-based risk score serves as an independent prognostic factor in OSA-related lung cancer

**Figure 4:**
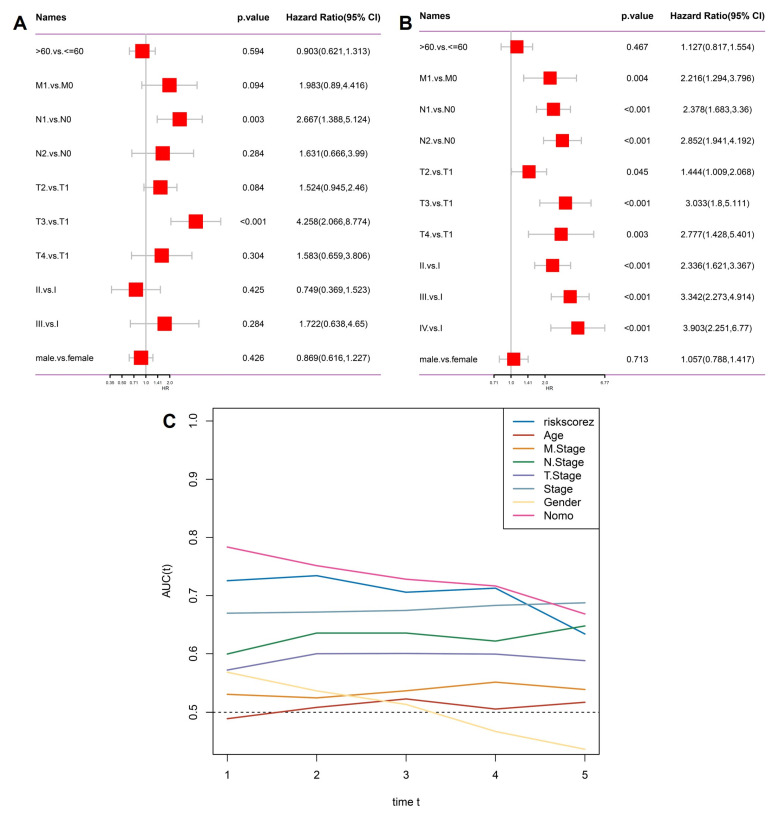
** Independent prognostic evaluation of the stemness signature.** (A) Multivariate Cox regression including clinical variables (age, gender, T stage, N stage, M stage, overall stage) and risk score. (B) Significant factors from Cox regression, highlighting stemness risk score alongside T stage and N stage as independent predictors. (C) Time-dependent AUC curves showing the predictive accuracy of the risk score compared with conventional clinical parameters.

To test whether the stemness-derived risk score could serve as an independent prognostic factor, we performed multivariate Cox regression analysis incorporating clinicopathological features (Figure 4A). The risk score remained significantly associated with overall survival even after adjustment for age, sex, and TNM stage, confirming its independence from traditional clinical parameters. Subsequently, we extracted only the significant predictors to construct a refined model (Figure 4B). In this reduced analysis, the stemness risk score, together with N stage and T stage, consistently emerged as robust prognostic indicators, whereas variables such as age and gender did not reach statistical significance. Time-dependent ROC analysis further demonstrated that the stemness risk score outperformed conventional variables in predictive accuracy, maintaining AUC values above 0.70 across multiple timepoints (Figure 4C). This stability underscores the clinical value of the model as a reliable risk stratification tool. Together, these findings establish the OSA-associated stemness signature as an independent prognostic factor with superior predictive performance compared to traditional staging systems, thereby providing a molecularly informed framework for patient management.

### 3.4 Stemness-high tumors display altered immune infiltration, checkpoint expression, and stromal remodeling

**Figure 5:**
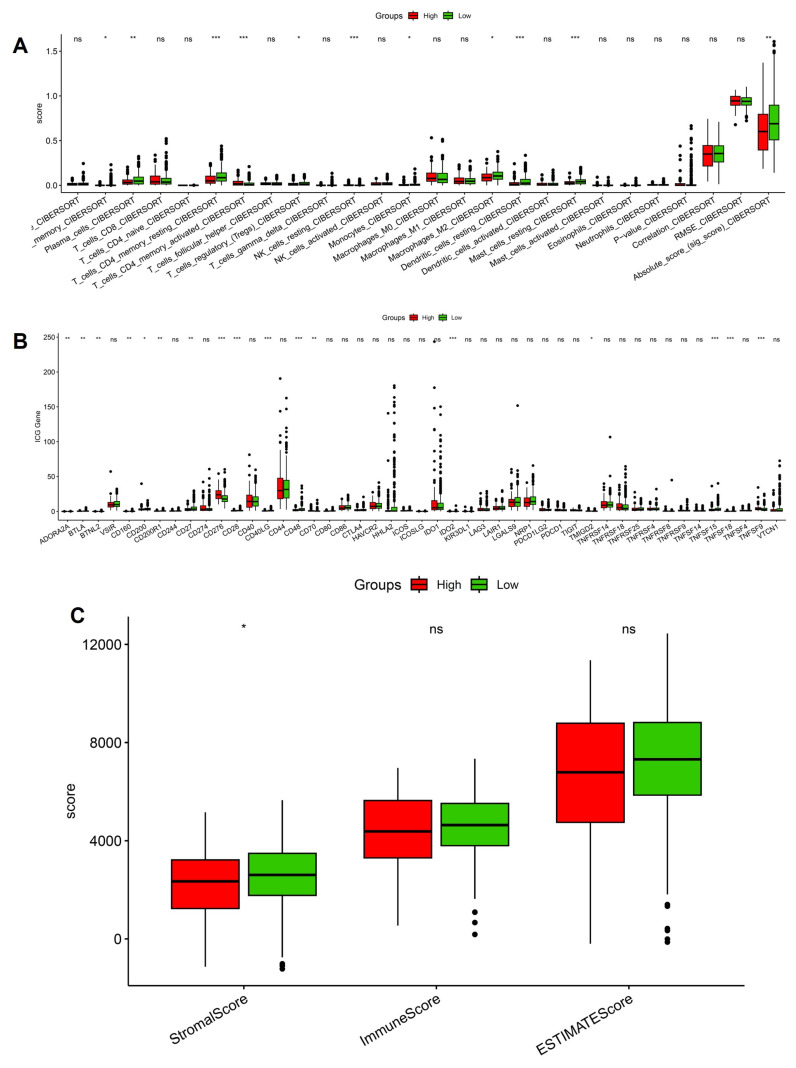
** Immune landscape differences between high- and low-stemness groups.** (A) CIBERSORT-based immune cell infiltration analysis showing altered abundance of T cells, macrophages, dendritic cells, and overall immune scores. (B) Expression of immune checkpoint genes compared between high- and low-stemness tumors. (C) Summary of stemness-immune associations.

We next investigated the immune and stromal correlates of stemness by integrating CIBERSORT, checkpoint profiling, and ESTIMATE analysis. CIBERSORT deconvolution revealed significant enrichment of immunoregulatory populations in stemness-high tumors, including activated CD4⁺ T cells, regulatory T cells, and M2-polarized macrophages, whereas low-stemness tumors displayed relatively higher fractions of naïve B cells and resting dendritic cells (Figure 5A). These shifts suggest that stemness programs not only promote effector activation but also skew the immune milieu toward an immunosuppressive phenotype dominated by Tregs and M2 macrophages. Consistent with this observation, checkpoint expression analysis demonstrated broadly elevated levels of inhibitory molecules in the stemness-high group, including PDCD1 (PD-1), CD274 (PD-L1), CTLA4, and LAG3 (Figure 5B). The coordinated upregulation of these checkpoints implies that stemness-driven tumors rely on multiple redundant inhibitory pathways to dampen T-cell activation and evade immune destruction. Interestingly, some costimulatory ligands were also variably increased, reflecting a mixed signaling environment that may initially activate but ultimately exhaust T-cell responses. ESTIMATE analysis further highlighted that stemness-high tumors harbored significantly higher stromal scores, while immune and composite scores did not differ markedly between groups (Figure 5C). This pattern suggests that stemness not only influences immune cell infiltration but also enhances stromal remodeling, potentially reinforcing physical and metabolic barriers to immune clearance. Notably, the immune infiltration patterns, checkpoint activation, and stromal remodeling observed in stemness-high human LUAD tumors are consistent with hypoxia-driven immune remodeling identified in the murine intermittent hypoxia model, supporting a coherent functional link between hypoxic stress, immune dysfunction, and tumor progression. Together, these data establish that OSA-associated stemness is intricately linked with an immunosuppressive and stromal-enriched tumor microenvironment. By coupling regulatory immune infiltration, checkpoint upregulation, and stromal expansion, stemness programs generate a tumor ecosystem optimized for immune escape and disease progression.

### 3.5 Stemness scores are elevated in tumors and associated with advanced clinical features in OSA-related lung cancer

**Figure 6:**
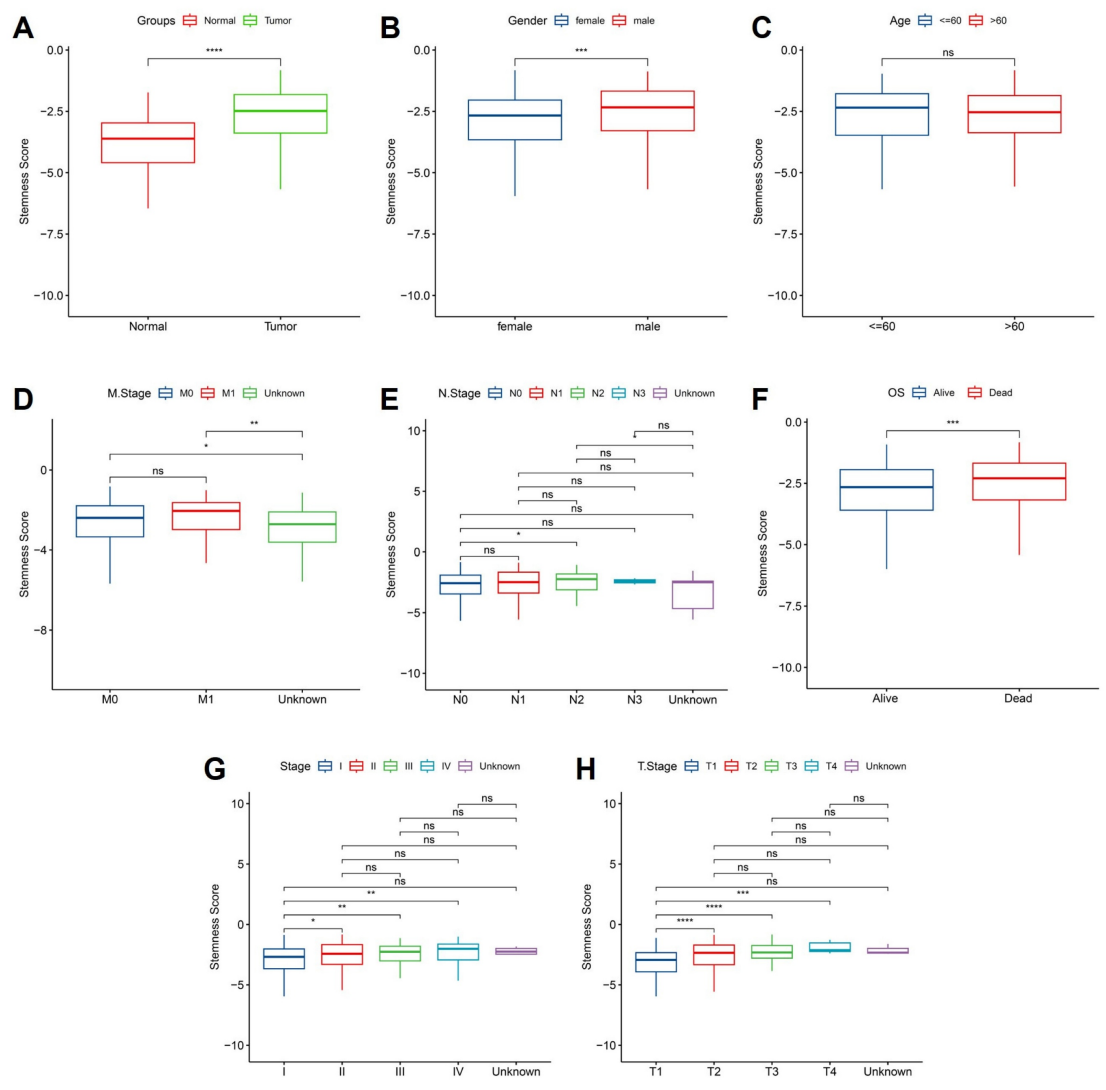
** Association of stemness scores with clinical features.** (A) Stemness was higher in tumors vs. normal tissues. (B) Male patients showed higher stemness than females. (C) No significant difference by age. (D) Metastatic (M1) cases exhibited higher stemness than M0. (E) No clear trend across lymph node stages. (F) High stemness correlated with worse overall survival. (G) Stemness increased with advanced clinical stage. (H) Stemness was elevated in higher T stages.

To further elucidate the clinical relevance of hypoxia-associated stemness programs, we examined stemness scores across patient subgroups stratified by clinicopathological features. Tumor tissues exhibited significantly elevated stemness compared with matched normal samples (Figure 6A), reinforcing the concept that intermittent hypoxia and OSA-driven microenvironmental stress favor acquisition of progenitor-like traits. This elevation was particularly evident in male patients (Figure 6B), suggesting sex-related differences in hypoxia response pathways, potentially mediated by hormonal or immunological factors. In contrast, patient age had no significant impact on stemness distribution (Figure 6C), highlighting that hypoxia-induced stemness remodeling is independent of chronological aging. Metastatic disease status was tightly linked to stemness reprogramming: patients with distant metastasis (M1) harbored significantly higher stemness scores than non-metastatic cases (M0) (Figure 6D). Interestingly, lymph node involvement (N stage) alone did not correlate strongly with stemness (Figure 6E), suggesting that systemic dissemination rather than regional spread is more tightly governed by stemness-driven biology. Importantly, survival analysis demonstrated that elevated stemness scores predicted significantly worse overall survival (Figure 6F), underscoring its prognostic utility. When stratified by pathological stage, stemness scores exhibited a progressive rise with advancing tumor stage (Figure 5G), with marked increases from stage I-II to III-IV, implicating stemness as a driver of aggressive disease evolution. Similarly, analysis of primary tumor burden (T stage) revealed a stepwise elevation in stemness from T1 to T3 (Figure 6H), consistent with its role in sustaining proliferation, therapy resistance, and local invasiveness. Together, these findings provide compelling evidence that hypoxia-associated stemness, initially identified in the single-cell analyses, translates into clinically measurable signals that track with tumor aggressiveness, metastatic spread, and poor survival outcomes. This strongly supports the hypothesis that OSA-mediated intermittent hypoxia not only remodels immune and epithelial compartments but also accelerates malignant progression through stemness-driven mechanisms. Targeting stemness-associated vulnerabilities may therefore represent a promising therapeutic strategy in OSA-related lung cancer.

### 3.6 Stemness enrichment is associated with immune checkpoint activation in OSA-related lung cancer

**Figure 7:**
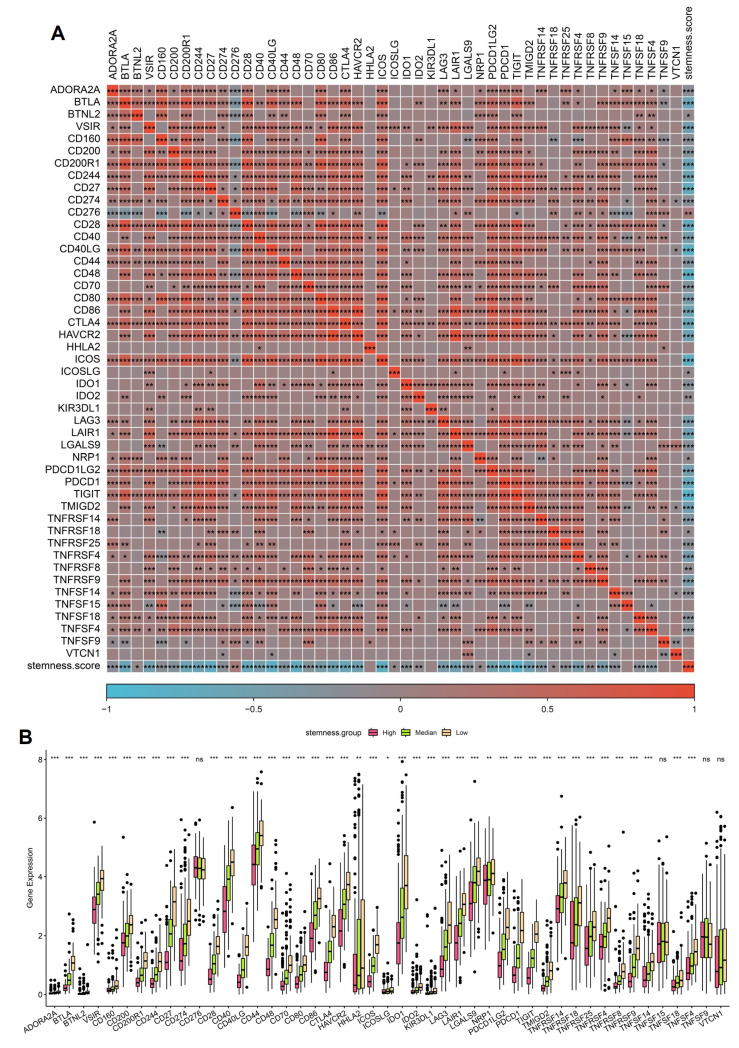
** Association of stemness scores with immune checkpoint expression.** (A) Correlation heatmap showing positive associations between stemness score and multiple immune checkpoint genes. (B) Boxplots comparing checkpoint gene expression across high, medium, and low stemness groups.

Given the prognostic impact of stemness, we next examined its interplay with immune checkpoint expression, a central determinant of tumor immune evasion and immunotherapy responsiveness. Correlation heatmap analysis revealed that high stemness scores were strongly associated with increased expression of classical immune checkpoints, including PDCD1 (PD-1), CD274 (PD-L1), CTLA4, LAG3, and TIGIT, as well as costimulatory ligands such as CD80/CD86 (Figure 7A). These findings suggest that stemness-high tumors adopt a transcriptional program that converges on immune checkpoint activation, enabling them to suppress T-cell effector responses and evade immune surveillance. Boxplot comparisons further demonstrated that tumors in the high-stemness group exhibited significantly elevated expression of most inhibitory checkpoint genes compared with medium- and low-stemness groups (Figure 7B). Notably, PD-1/PD-L1, CTLA4, and LAG3 showed the most pronounced differences, indicating that stemness-enriched tumors may be particularly dependent on checkpoint-mediated immunosuppression. In contrast, only a limited subset of checkpoint molecules displayed no significant differences across stemness strata, highlighting the specificity of this association. These findings underscore a critical mechanistic link: intermittent hypoxia in OSA may foster stemness programs that not only drive tumor aggressiveness but also co-opt immune checkpoint pathways to facilitate immune evasion. Clinically, this dual effect provides a rationale for stratifying OSA-related lung cancer patients by stemness score to predict immunotherapy response. Patients with high stemness tumors may derive greater benefit from checkpoint inhibitor therapies.

### 3.7 High stemness predicts stronger immune evasion potential by TIDE in OSA-related lung cancer

**Figure 8:**
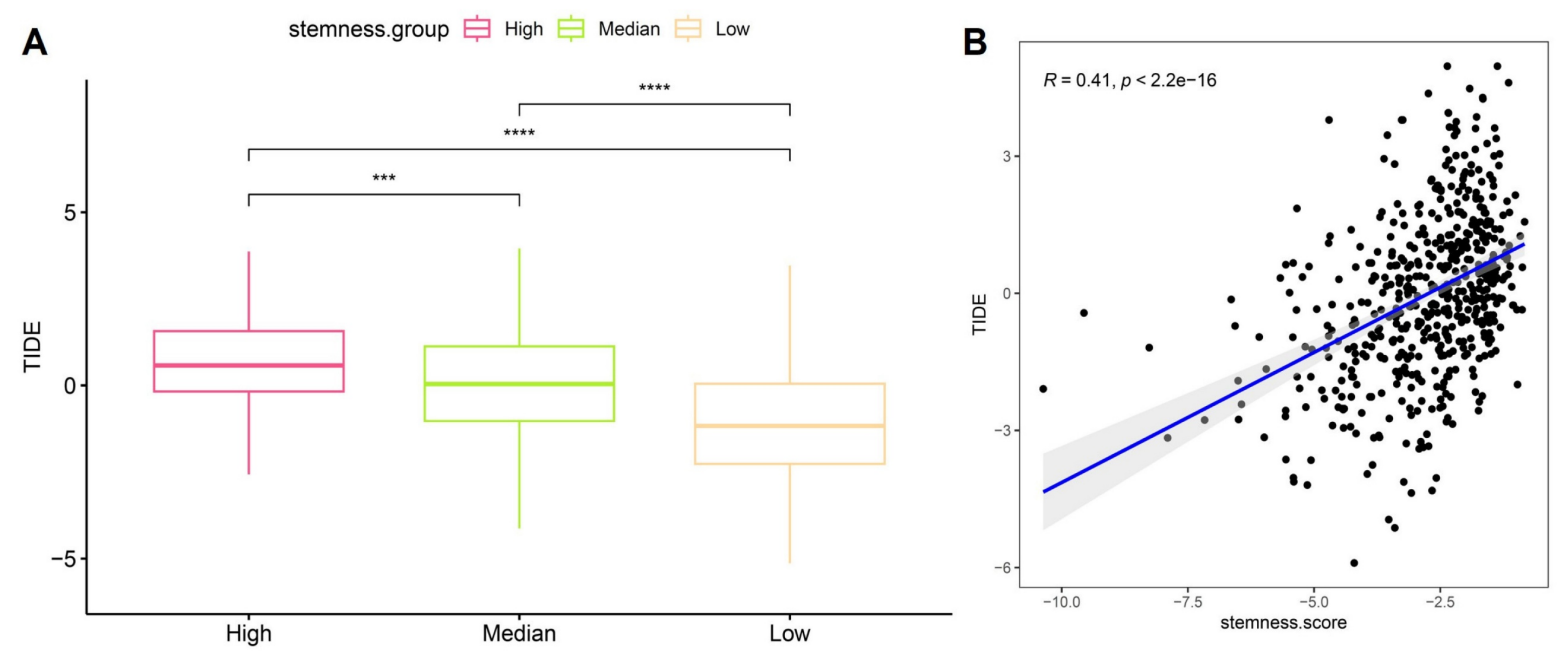
** Association of stemness with TIDE scores.** (A) Boxplots showing significantly higher TIDE scores in high-stemness tumors compared with medium- and low-stemness groups. (B) Positive correlation between stemness scores and TIDE values (R = 0.41, p < 2.2e-16).

To evaluate whether stemness signatures influence tumor immune escape, we integrated Tumor Immune Dysfunction and Exclusion (TIDE) analysis with stemness stratification. Tumors classified as high-stemness exhibited markedly elevated TIDE scores compared to medium- and low-stemness groups (Figure 8A), indicating enhanced likelihood of immune evasion. This trend persisted across independent comparisons, underscoring the robustness of the association. Linear correlation analysis further confirmed a significant positive relationship between stemness score and TIDE value (R = 0.41, p < 2.2e-16) (Figure 8B), suggesting that stemness-enriched tumors consistently harbor stronger immunosuppressive potential. Mechanistically, the link between stemness and TIDE likely reflects synergistic interactions between progenitor-like transcriptional states, mitochondrial stress programs, and checkpoint activation identified before. High-stemness tumors appear to adopt a metabolic and transcriptional profile that not only promotes self-renewal and resistance to differentiation but also fosters an immune-excluded microenvironment, thereby evading cytotoxic T-cell surveillance. These dual features may explain why OSA-related hypoxia accelerates both malignant progression and immune resistance in lung cancer. Clinically, these findings position stemness as a potential biomarker for predicting immunotherapy responsiveness in OSA-associated lung cancer. Patients with high-stemness tumors may be less likely to benefit from immune checkpoint blockade alone and could require combinational strategies targeting stemness pathways to overcome adaptive immune resistance.

### 3.8 Single-cell transcriptomic landscape reveals immune cell heterogeneity in OSA-associated lung tissue

**Figure 9:**
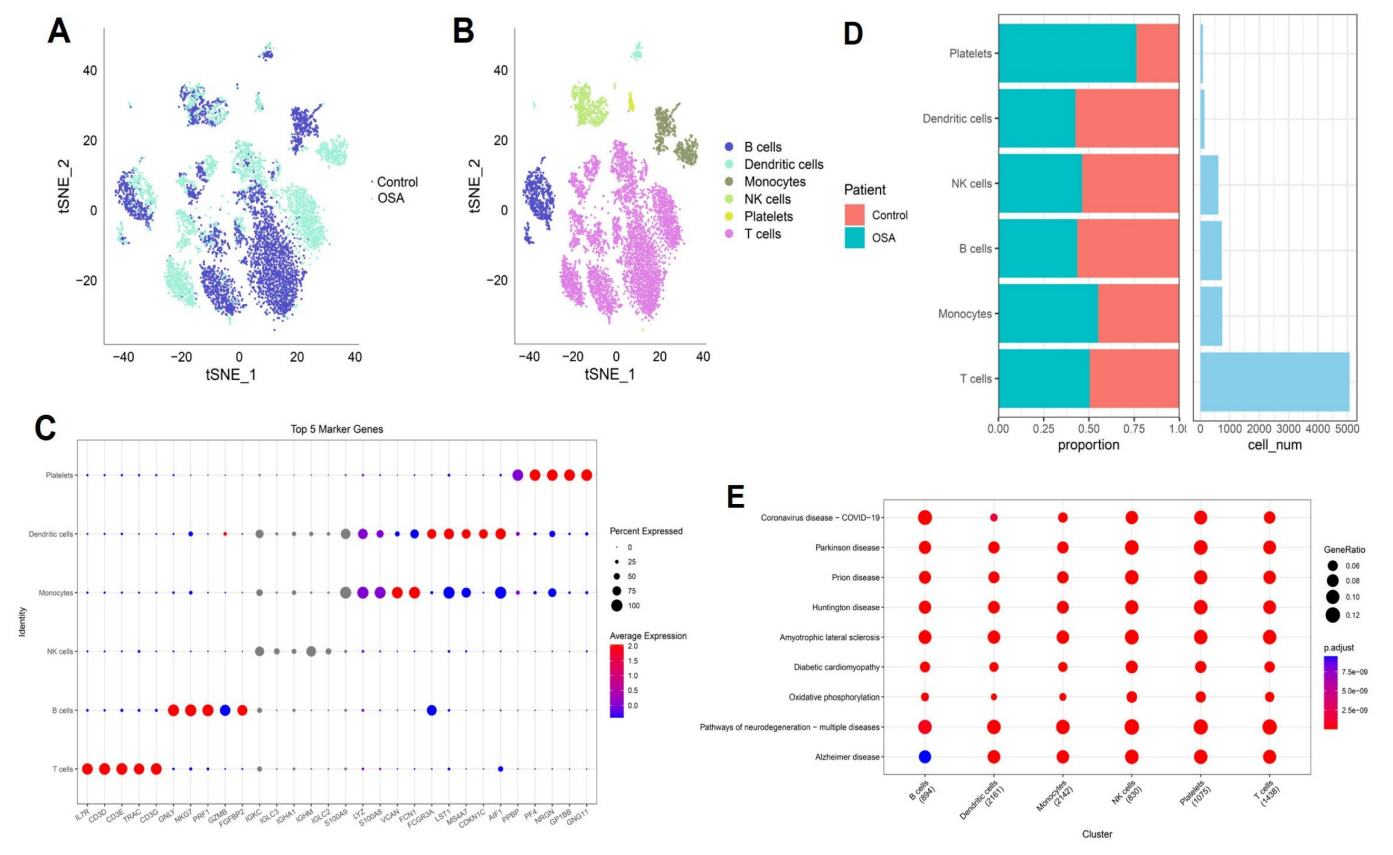
** Immune landscape of OSA and control lungs.** (A) t-SNE plots showing global cell distribution (OSA vs. control). (B) Annotation of six immune lineages. (C) Bubble plot of top marker genes per lineage. (D) Proportional changes in immune subsets. (E) Pathway enrichment across clusters.

Single-cell transcriptomic profiling revealed profound reshaping of the lung immune microenvironment in response to OSA-associated intermittent hypoxia. Dimensionality reduction analyses demonstrated clear segregation of OSA and control cells, reflecting condition-specific transcriptional programs rather than technical artifacts. Within this landscape, we identified six predominant immune populations, including T cells, B cells, NK cells, monocytes, dendritic cells, and platelets, each defined by canonical lineage-specific markers such as CD3D for T cells, MS4A1 for B cells, NKG7 for NK cells, and FCGR3A for monocytes. Notably, T cells and monocytes comprised the largest fractions in OSA lungs, consistent with their central role in hypoxia-driven inflammation. Quantitative comparisons revealed a disproportionate expansion of effector T cells and pro-inflammatory monocytes, with a concomitant reduction in dendritic cells and B cells. This shift suggests a skewing toward cytotoxic and innate effector programs, potentially at the expense of antigen-presenting and humoral immune functions. Such remodeling aligns with previous observations in OSA patients, where intermittent hypoxia enhances systemic inflammation, disrupts adaptive immunity, and promotes an immunosuppressive milieu favorable for tumor initiation. The observed expansion of platelets under hypoxia further underscores the interplay between coagulation, chronic inflammation, and tumor microenvironmental priming. Pathway enrichment analyses provided mechanistic insights into these cellular shifts. Across immune subsets, we detected enrichment in oxidative phosphorylation, mitochondrial dysfunction, and neurodegenerative disease-related pathways, reflecting the systemic metabolic burden imposed by intermittent hypoxia. Importantly, the upregulation of mitochondrial stress signatures in T cells and monocytes may amplify reactive oxygen species (ROS) production, fueling DNA damage and oncogenic signaling cascades. Simultaneous enrichment in viral infection and inflammatory signaling pathways highlights an activated, stress-responsive state that could facilitate both tissue injury and immune evasion. Taken together, these findings suggest that OSA-driven intermittent hypoxia orchestrates a dual-edged immune reprogramming process: on one hand, amplifying effector activity and metabolic stress in monocytes and T cells; on the other, diminishing antigen presentation and B-cell-mediated surveillance. Such an imbalance may establish a chronic inflammatory and metabolically perturbed lung environment conducive to fibrosis, epithelial-to-mesenchymal transition, and ultimately, malignant transformation. This immune remodeling provides a plausible mechanistic link between OSA and increased lung cancer susceptibility, and establishes a foundation for exploring immunometabolic vulnerabilities in OSA-related oncogenesis (Figure 9). Importantly, the single-cell RNA-seq dataset was derived from non-malignant murine lung tissue exposed to intermittent hypoxia. Therefore, epithelial-like populations identified in this analysis are interpreted as hypoxia-responsive epithelial states rather than premalignant or early neoplastic entities. The observed transcriptional changes reflect hypoxia-induced cellular plasticity, not direct evidence of tumor initiation.

### 3.9 CytoTRACE analysis highlights stemness heterogeneity and differentiation plasticity under OSA-associated hypoxia

**Figure 10:**
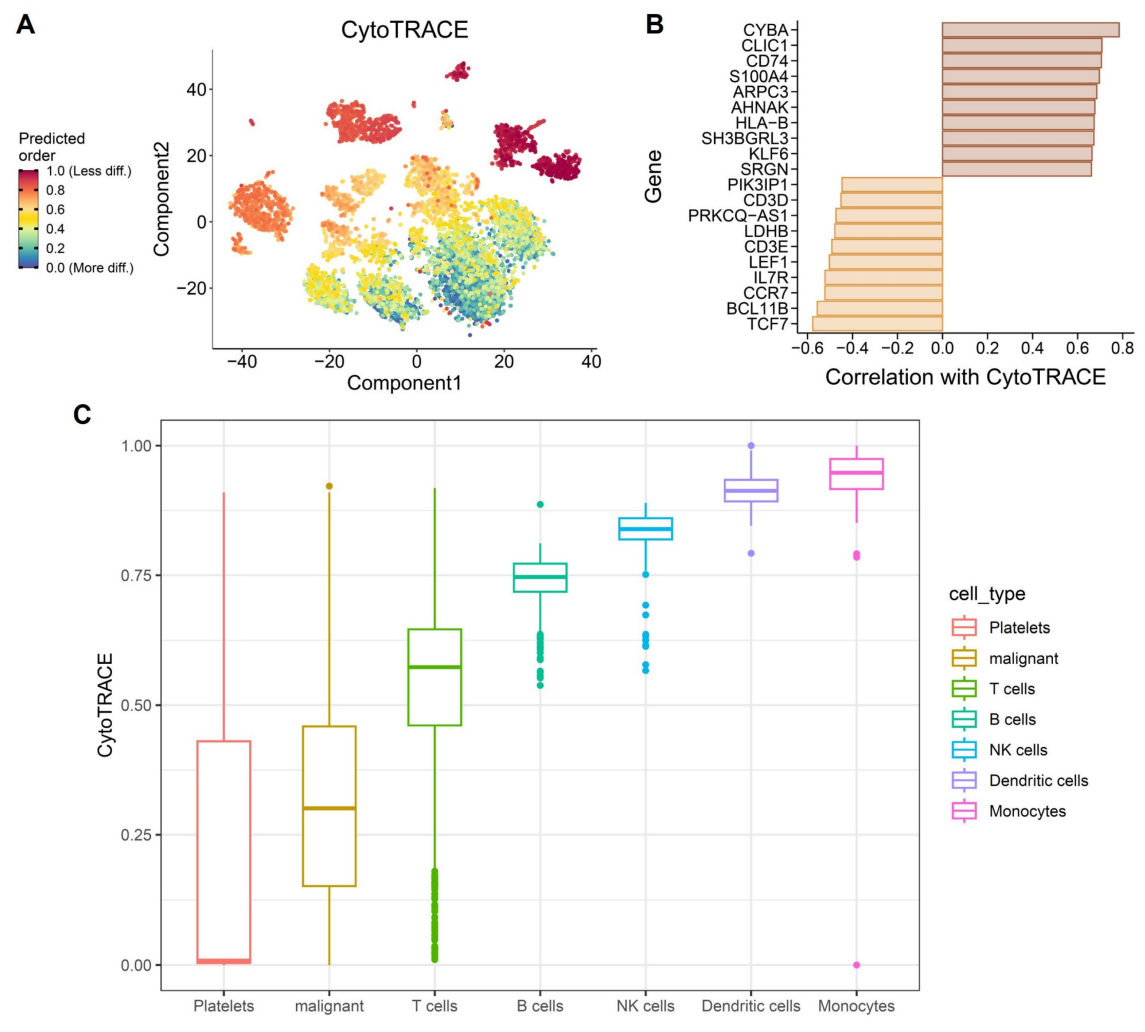
** CytoTRACE-based evaluation of differentiation plasticity in immune and epithelial populations under OSA-associated intermittent hypoxia.** (A) CytoTRACE visualization of single-cell transcriptomes, with colors representing predicted differentiation potential (red = less differentiated, blue = more differentiated). (B) Correlation of CytoTRACE scores with gene expression, showing positive associations with stemness-related regulator such as, TCF7, BCL11B, IL7R, and CCR7 and negative correlations with metabolic or stress-response genes including LDHB, and PRKCQ-AS1. (C) Boxplots of CytoTRACE scores across cell types, indicating higher stemness like signatures in monocytes, dendritic cells, and NK cells, while platelets and epithelial-like cell populations exhibited reduced differentiation potential. All cell populations shown are derived from non-malignant lung tissue and reflect hypoxia-induced differentiation plasticity rather than malignant transformation.

To further characterize how obstructive sleep apnea-related intermittent hypoxia shapes cellular differentiation dynamics in the lung, we applied CytoTRACE to infer stemness across immune and epithelial cell populations in the single-cell dataset. As these analyses were performed using non-malignant murine lung tissue, CytoTRACE scores are interpreted as reflecting hypoxia-induced differentiation plasticity rather than intrinsic tumor stemness or malignant transformation. The analysis revealed striking heterogeneity in differentiation potential (Figure 10A), with subsets of monocytes, dendritic cells, and NK cells displaying high CytoTRACE scores, indicative of preserved stemness and plasticity, whereas platelets and epithelial-like cell populations exhibited lower scores, suggestive of terminal or stress-associated differentiation states. Such divergent differentiation landscapes underscore the impact of hypoxic stress on remodeling the immune and epithelial compartments of the lung. Gene-level correlations further illuminated transcriptional programs associated with stemness. Positive correlations included canonical T cell development and survival factors (TCF7, BCL11B, IL7R, CCR7), as well as regulators of progenitor-like states such as LEF1 and CD3E (Figure 10B). Conversely, negative correlations with metabolic genes (LDHB) and hypoxia-responsive factors (PRKCQ-AS1, SRGN) indicate that mitochondrial dysfunction and stress responses antagonize differentiation potential, reinforcing the theme of mito-nuclear imbalance. This dual signature suggests that intermittent hypoxia simultaneously maintains progenitor-like traits in certain immune subsets while driving metabolic exhaustion in others. At the population level, CytoTRACE stratification demonstrated that monocytes and dendritic cells harbored the highest stemness potential (Figure 10C), consistent with their roles as plastic innate effectors capable of fueling inflammatory remodeling and antigen presentation under stress. NK cells also retained stemness signatures, which may support cytotoxic adaptability but could also contribute to immune dysregulation in a hypoxic microenvironment. In contrast, epithelial-like cell populations displayed low CytoTRACE scores, indicating impaired differentiation and a shift toward metabolic rigidity. This phenotype may reflect the combined impact of intermittent hypoxia on mitochondrial dysfunction and chromatin remodeling, fostering conditions conducive to malignant persistence and resistance to apoptosis. Collectively, these findings highlight a paradoxical role of OSA-driven hypoxia in lung tissues: while it preserves stemness and plasticity in immune populations that may perpetuate chronic inflammation, it simultaneously enforces differentiation arrest and metabolic maladaptation in malignant cells. Such reciprocal programming may create a tumor-promoting ecosystem, where immune cell plasticity supports a pro-inflammatory and pro-angiogenic niche, while malignant cells capitalize on hypoxia-induced metabolic rewiring to evade immune surveillance. This stemness imbalance provides a mechanistic bridge between OSA pathophysiology and lung cancer progression, suggesting that targeting hypoxia-related stemness pathways could represent a therapeutic avenue. These stemness-associated features reflect altered differentiation states induced by hypoxic stress and should not be interpreted as evidence of malignant transformation.

### 3.10 Stemness-based risk groups predict differential responses to immunotherapy

**Figure 11:**
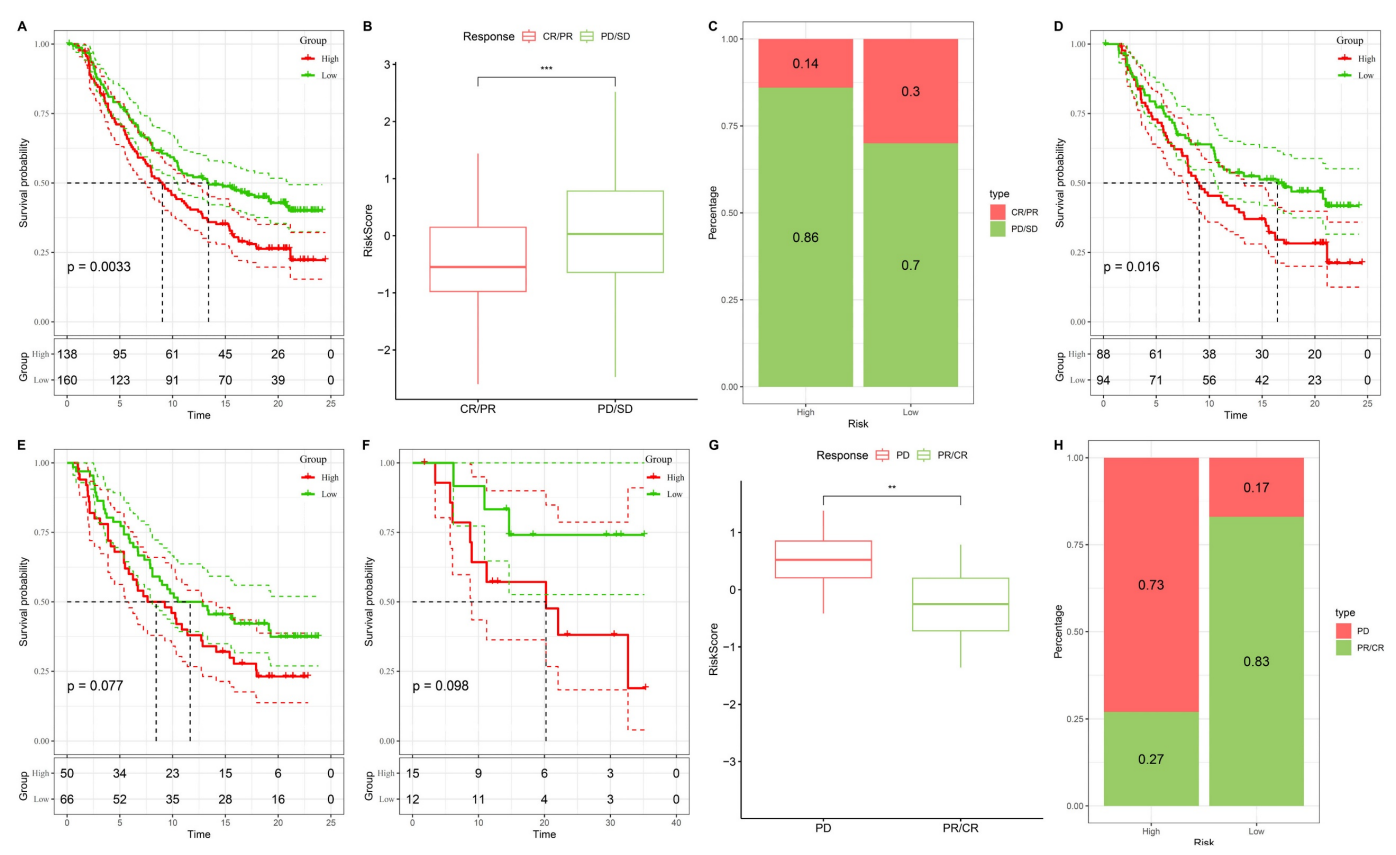
** Validation of the stemness-based risk model in immunotherapy datasets (IMvigor210 and GSE78820).** (A) Kaplan-Meier survival analysis of IMvigor210 showing significantly shorter survival in the high-risk group compared with the low-risk group. (B) Boxplot analysis of IMvigor210 demonstrating that patients with progressive disease (PD) or stable disease (SD) had higher risk scores than those with complete response (CR) or partial response (PR). (C) Distribution of treatment responses in IMvigor210, with the low-risk group enriched for CR/PR and the high-risk group enriched for PD/SD. (D) Kaplan-Meier survival analysis of GSE78820 showing inferior survival outcomes in high-risk patients. (E) Subgroup Kaplan-Meier validation in GSE78820 confirming a trend toward reduced survival in the high-risk group. (F) Additional survival validation in a smaller subset of GSE78820, again demonstrating poorer outcomes in high-risk patients. (G) Boxplot analysis of GSE78820 showing higher risk scores in non-responders (PD) compared with responders (CR/PR). (H) Distribution of treatment responses in GSE78820 indicating that the majority of low-risk patients derived clinical benefit (CR/PR), whereas high-risk patients were predominantly non-responders.

To further evaluate the predictive value of the stemness-based risk model in the context of immunotherapy, we applied the model to two independent ICB-treated cohorts, IMvigor210 and GSE78820. In the IMvigor210 cohort, Kaplan-Meier survival analysis revealed that patients in the high-risk group had significantly shorter overall survival compared with those in the low-risk group (Figure 11A). When stratified by treatment response, non-responders (progressive disease [PD] or stable disease [SD]) exhibited substantially higher risk scores than responders (complete response [CR] or partial response [PR]) (Figure 11B). Consistent with these findings, response distribution analysis showed that clinical benefit (CR/PR) was enriched in the low-risk group, whereas the high-risk group was dominated by PD/SD cases (Figure 11C). Similar trends were observed in the GSE78820 immunotherapy cohort. Kaplan-Meier survival curves consistently demonstrated inferior outcomes in high-risk patients compared with their low-risk counterparts (Figure 11D-F). Boxplot comparisons further indicated that non-responders carried higher risk scores than responders, supporting the robustness of the model across different patient populations (Figure 11G). Finally, response distribution analysis confirmed that the low-risk group was enriched for CR/PR cases, whereas the high-risk group was predominantly composed of non-responders (Figure 11H). Taken together, these results demonstrate that the stemness-based risk score not only stratifies prognosis but also serves as a predictive biomarker for immunotherapy efficacy. Patients in the low-risk group are more likely to achieve durable clinical benefit from immune checkpoint blockade, while high-risk patients, characterized by stemness-associated immune evasion, exhibit limited responsiveness. These findings suggest that integrating stemness stratification into clinical decision-making may help identify patients most likely to benefit from immunotherapy and guide the development of rational combination strategies for high-risk individuals.

### 3.11 Immunohistochemical validation of stemness-associated genes in LUAD

**Figure 12:**
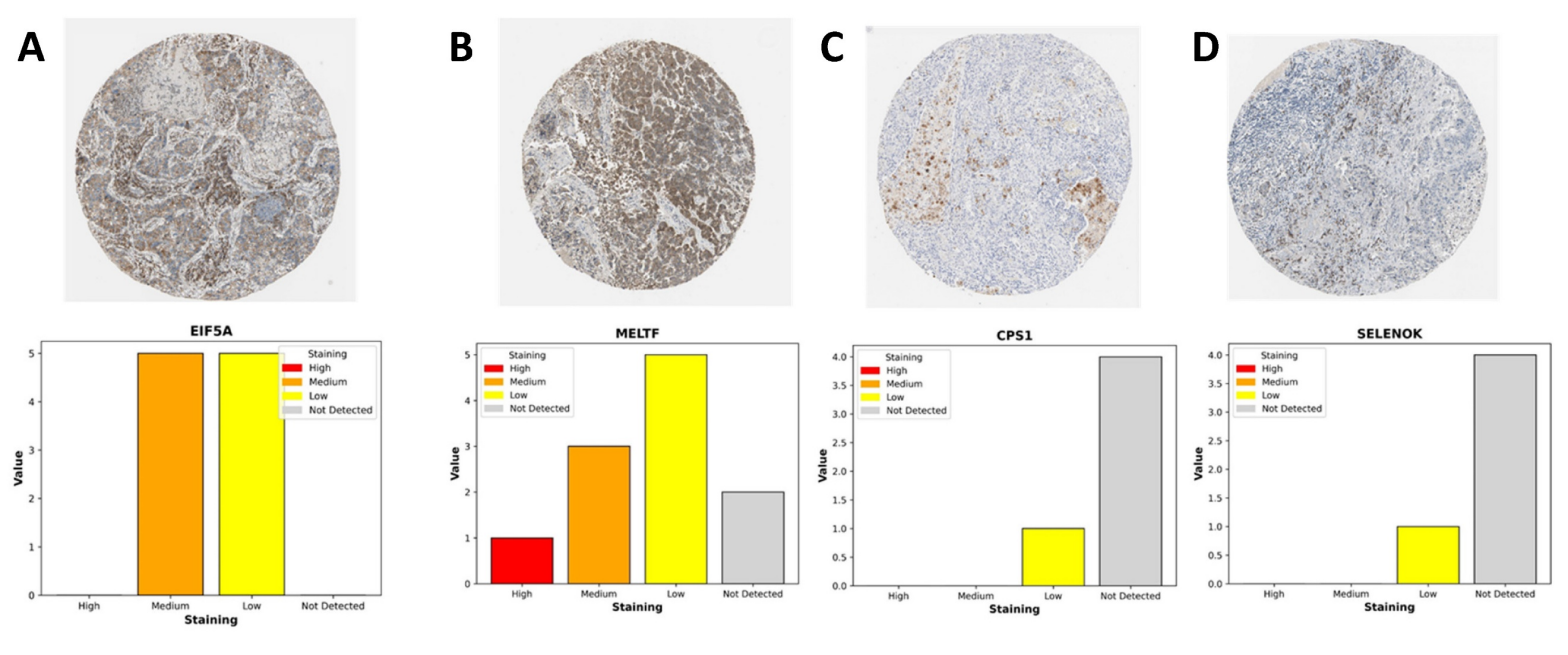
** Immunohistochemical expression of LUAD-associated genes from the Human Protein Atlas.** (A) EIF5A: Moderate-to-strong cytoplasmic and nuclear staining observed in malignant epithelial cells, consistent with its role in sustaining translational activity and proliferation.(B) MELTF: Predominantly membranous and cytoplasmic staining enriched in tumor cells, highlighting its involvement in iron metabolism and hypoxia-driven redox regulation.(C) CPS1: Weak or absent cytoplasmic staining, reflecting suppression of urea cycle activity and mitochondrial metabolism in LUAD.(D) SELENOK: Minimal staining in most tumor samples, suggesting downregulation of antioxidant and ER-associated homeostasis functions. Bar plots summarize staining intensity categories (high, medium, low, not detected) across LUAD patient samples.

To complement our transcriptomic analyses, we next assessed the protein-level expression of representative genes from the stemness-associated signature using immunohistochemistry (IHC) data from the Human Protein Atlas (Figure 12). Consistent with the transcriptional profiles, EIF5A and MELTF displayed prominent staining in LUAD tissues, whereas CPS1 and SELENOK exhibited weak or undetectable expression in most samples. EIF5A showed moderate-to-strong cytoplasmic and nuclear staining in malignant epithelial cells, reflecting its role as a translation initiation factor that supports proliferative and metabolic programs in stemness-high tumors. MELTF, a transferrin receptor homolog involved in iron metabolism and cellular redox balance, demonstrated enriched membranous and cytoplasmic staining, further supporting its function in metabolic reprogramming under hypoxic and stemness-driven conditions. By contrast, CPS1, a key enzyme in the urea cycle, and SELENOK, a regulator of ER-associated protein degradation and redox homeostasis, were largely absent or only weakly expressed in tumor sections. The downregulation of these metabolic regulators is consistent with our transcriptomic findings that stemness-high tumors suppress mitochondrial and antioxidant pathways to favor proliferative adaptability. Quantitative summaries of IHC scoring revealed that the majority of LUAD samples exhibited medium-to-high staining for EIF5A and MELTF, while CPS1 and SELENOK were predominantly categorized as low or not detected. This expression pattern highlights a functional dichotomy: stemness-promoting genes (EIF5A, MELTF) are selectively upregulated at the protein level, whereas metabolic homeostasis genes (CPS1, SELENOK) are suppressed, reinforcing the hypothesis that OSA-associated hypoxia drives a shift toward translational and iron-regulated growth programs while diminishing metabolic resilience. Together, these IHC findings provide orthogonal validation of our multi-omics model, confirming that stemness-associated risk genes are differentially regulated at the protein level in LUAD tissues. This reinforces the biological plausibility of our stemness-based prognostic signature and further illustrates how intermittent hypoxia-driven reprogramming converges on both translational and metabolic axes to support tumor progression.

### 3.12 Stemness-based risk groups exhibit differential drug sensitivity profiles

**Figure 13:**
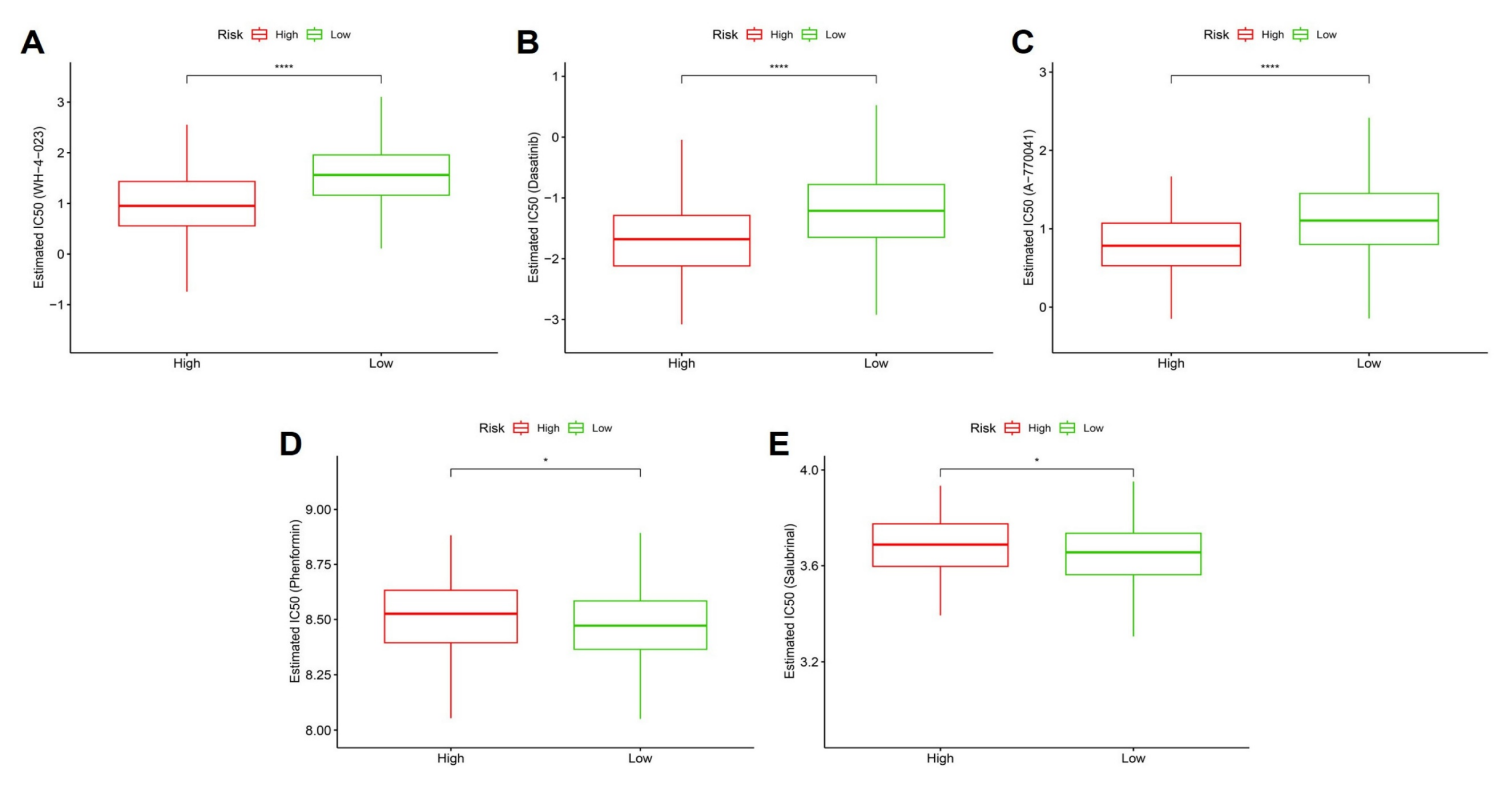
** Predicted drug responses in high- vs. low-risk groups.** (A-C) High-risk tumors showed significantly lower estimated IC50 values for WH-4023, dasatinib, and A-770041, suggesting higher sensitivity. (D-E) High-risk tumors displayed decreased sensitivity to phenformin and salubrinal.

To investigate whether the stemness-derived risk signature could inform therapeutic decision-making, we evaluated drug sensitivity patterns between high- and low-risk groups using estimated IC50 values. High-risk tumors consistently demonstrated significantly lower IC50 values for WH-4023, dasatinib, and A-770041 (Figure 13A-C), indicating enhanced susceptibility to these targeted agents. These drugs primarily act on tyrosine kinase and T-cell signaling pathways, suggesting that stemness-driven tumors may be particularly vulnerable to interventions disrupting oncogenic signaling and immune regulation. Similarly, metabolic and stress-response modulators, including phenformin (a mitochondrial complex I inhibitor) and salubrinal (an ER stress modulator), also showed stronger predicted efficacy in the high-risk group (Figure 13D-E). This aligns with prior observations that stemness-high tumors harbor mitochondrial dysfunction and heightened stress responses, rendering them more sensitive to compounds targeting bioenergetic and proteostasis vulnerabilities. Taken together, these findings highlight that OSA-associated stemness not only drives tumor aggressiveness and immune evasion but also defines a distinct therapeutic vulnerability landscape. Patients stratified into the high-risk group may benefit from tailored regimens incorporating kinase inhibitors and metabolic modulators, whereas low-risk patients may require alternative therapeutic strategies. This integration of stemness biology with drug response prediction underscores the translational value of the model in guiding personalized therapy.

## 4. Discussion

This study provides novel insights into the biological link between obstructive sleep apnea (OSA), intermittent hypoxia (IH), and lung cancer progression. By integrating single-cell RNA sequencing, bulk transcriptomics, and computational modeling, we demonstrate that IH is associated with stemness like transcriptional plasticity, mitochondrial dysfunction, and immune remodeling, ultimately contributing to a tumor-promoting microenvironment. These findings complement prior epidemiological evidence linking OSA severity with lung cancer risk and poor outcomes, while offering molecular and cellular context for this association. At the cellular level, our scRNA-seq analysis revealed that IH is accompanied by marked reshaping of the lung immune landscape. Expansion of effector T cells and pro-inflammatory monocytes reflects an activated immune state, whereas concomitant enrichment of regulatory T cells and M2 macrophages suggests a parallel immunosuppressive shift 62, 63. This duality reflects a well-recognized paradox in cancer biology, wherein chronic inflammation activation promotes tissue remodeling and genomic stress, while immunosuppressive cell populations dampen effective antitumor immunity 64. Importantly, our CytoTRACE analysis highlighted that IH is associated with stemness-like differentiation plasticity in immune cell populations, sustaining progenitor-like features in monocytes and dendritic cells while epithelial-like cells exhibited stress-associated differentiated states 65. These findings are consistent with hypoxia-driven activation of HIF-1α and c-Myc pathways, which are known to regulate both stemness and immunometabolism 66. Notably, as the single-cell analyses were conducted in non-malignant lung tissue, the stemness-associated features observed here are best interpreted as hypoxia-induced cellular plasticity rather than direct evidence of neoplastic transformation. From a metabolic perspective, stemness-high tumors exhibited a striking mito-nuclear imbalance, characterized by downregulation of mitochondrial transcripts and compensatory upregulation of nuclear-encoded oxidative phosphorylation genes 67. This imbalance may amplify reactive oxygen species (ROS) production, DNA damage, and activation of stress-responsive transcriptional programs, thereby potentially facilitating tumor progression and therapeutic resistance 68. These results expand upon prior reports linking intermittent hypoxia to mitochondrial fragmentation and dysfunction, suggesting that metabolic rewiring is a central axis by which OSA promotes oncogenesis 69.

Clinically, the stemness-derived prognostic model demonstrated robust and independent predictive power across TCGA-LUAD and GSE31210 cohorts. Elevated stemness scores were associated with advanced tumor stage, metastatic dissemination, and inferior overall survival, consistent with the established role of stemness in tumor aggressiveness and therapy resistance. Beyond prognostication, our analyses revealed that stemness stratification informs therapeutic responsiveness. High-stemness tumors consistently exhibited immune checkpoint upregulation, elevated TIDE scores, and reduced responsiveness to checkpoint blockade, reflecting a stemness-driven immune-evasive phenotype 70. These findings resonate with emerging clinical data showing that stemness signatures predict poor response to immunotherapy across multiple cancer types, including NSCLC and melanoma 24, 71. Our findings support a coherent data-informed mechanistic model in which OSA-associated intermittent hypoxia functions as an upstream microenvironmental stressor that initiates immune and epithelial plasticity. Hypoxia-induced metabolic stress and mitochondrial dysfunction promote inflammatory activation while simultaneously favoring the expansion of immunosuppressive cell populations and immune checkpoint expression. This altered immune landscape creates permissive conditions for stemness-associated tumor programs, which in human LUAD cohorts manifest as immune evasion, reduced immunotherapy responsiveness, and adverse clinical outcomes. From a therapeutic standpoint, our drug sensitivity analysis suggests potential vulnerabilities within stemness-high tumors. Despite immune evasion, high-stemness tumors exhibited heightened sensitivity to kinase inhibitors (dasatinib, A-770041) and metabolic stress modulators (phenformin, salubrinal) 72, 73. This pattern is consistent with the concept of collateral vulnerabilities, whereby stemness-associated programs impose exploitable metabolic and signaling dependencies 74. Combination strategies targeting both stemness-associated pathways and immune checkpoints may therefore offer synergistic benefits. For example, dasatinib has been shown to modulate T-cell signaling and suppress hypoxia-induced oncogenic pathways, while Phenformin selectively targets tumors with mitochondrial dysfunction. Such approaches could be particularly valuable in OSA patients, who may harbor biologically distinct tumors with high stemness and poor immunotherapy response 75.

Our findings also carry broader implications for the field of sleep medicine and oncology. The demonstration that IH imprints a durable stemness and immune-escape phenotype in the lung suggests that OSA should be considered not only a comorbidity but also a cancer risk modifier 12. This raises the possibility that early diagnosis and treatment of OSA, for example with continuous positive airway pressure (CPAP), may attenuate tumor-promoting pathways. Indeed, clinical studies have shown that CPAP therapy reduces systemic inflammation and oxidative stress, though its impact on cancer incidence remains to be established. Future prospective studies integrating sleep phenotyping with molecular tumor profiling will be critical to validate whether OSA treatment modifies cancer risk and therapeutic response 76, 77.

Several limitations warrant consideration. This study primarily relies on computational analyses and publicly available transcriptomic datasets, and although findings were validated across independent human cohorts, experimental validation will be necessary to establish direct causal mechanisms. In addition, the use of murine intermittent hypoxia models and the absence of clinical OSA annotation in LUAD datasets may limit direct translational inference, underscoring the need for prospective, mechanistic studies.

In summary, this study identifies stemness as a key biological and clinical bridge linking OSA-associated intermittent hypoxia to lung cancer progression, Immune evasion, and therapeutic resistance. By integrating single-cell analysis, prognostic modeling, immune landscape profiling, and drug sensitivity prediction, we provide a comprehensive framework that advances understanding of OSA as an oncogenic risk factor. More importantly, our findings highlight actionable vulnerabilities and suggest that integrating stemness-targeted interventions with immunotherapy may improve outcomes in OSA-associated lung cancer, paving the way for precision oncology strategies tailored to patients with sleep-disordered breathing.

## 5. Conclusions

**Figure 14:**
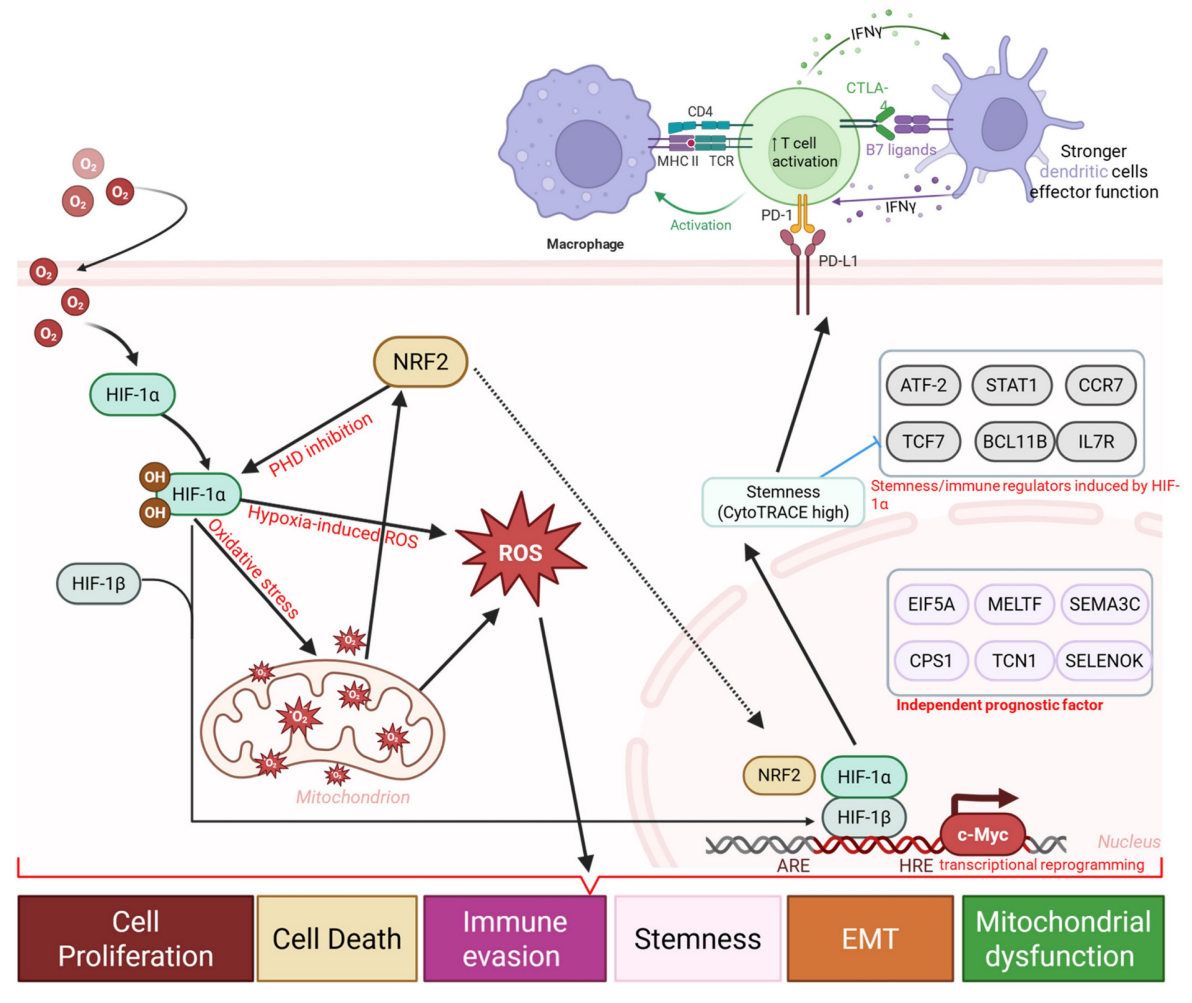
** Hypoxia-driven immune and stemness remodeling in OSA-associated lung cancer.** Schematic diagram illustrating a proposed mechanistic framework linking obstructive sleep apnea (OSA)-associated intermittent hypoxia to immune dysfunction and tumor progression. Intermittent hypoxia stabilizes HIF-1α and induces oxidative stress, and metabolic stress, leading to mitochondrial dysfunction, and transcriptional reprogramming. These changes promote immune and epithelial plasticity, enrichment of stemness-associated programs, and upregulation of immune checkpoints (PD-1, PD-L1, CTLA-4, B7 ligands), accompanied by T cell activation followed by exhaustion, altered dendritic cell function, and macrophage polarization.

In conclusion, (Figure 14) this integrative single-cell and transcriptomic study provides evidence that obstructive sleep apnea-associated intermittent hypoxia is linked to lung cancer progression through stemness -associated transcriptional plasticity, metabolic dysregulation, and immune remodeling. We demonstrate that stemness-high tumors are characterized by mitochondrial stress-related transcriptional programs, enrichment of developmental signaling pathway, immune checkpoint upregulation, and adverse clinical outcomes, while also exhibiting predicted vulnerabilities to selected kinase inhibitors and metabolic modulators. The stemness-based prognostic model developed in this study was validated across independent cohorts and identified as an independent indicator of patient survival and predicted immunotherapy responsiveness. These findings support stemness as a mechanistic and clinically relevant axis connecting OSA-associated hypoxic stress with tumor aggressiveness, highlight its potential utility as a prognostic biomarker and stratification factor. Future prospective studies integrating clinical OSA phenotyping, patient-derived tumor models, and functional validation will be essential to confirm these observations and refine precision oncology strategies for patients with OSA-related lung cancer.

## Abbreviations

OSA: Obstructive sleep apnea
IH: Intermittent hypoxia
NSCLC: Non-small cell lung cancer
LUAD: Lung adenocarcinoma
scRNA-seq: Single-cell RNA sequencing
UMAP: Uniform manifold approximation and projection
t-SNE: t-distributed stochastic neighbor embedding
CytoTRACE: Cellular Trajectory Reconstruction Analysis using gene Counts and Expression
TCGA: The Cancer Genome Atlas
GEO: Gene Expression Omnibus
DEG: Differentially expressed gene
GO: Gene Ontology
KEGG: Kyoto Encyclopedia of Genes and Genomes
ROC: Receiver operating characteristic
AUC: Area under the curve
Cox: Proportional hazards regression
LASSO: Least absolute shrinkage and selection operator
CIBERSORT: Cell type Identification by Estimating Relative Subsets of RNA Transcripts
ESTIMATE: Estimation of STromal and Immune cells in MAlignant Tumor tissues using Expression data
TIDE: Tumor Immune Dysfunction and Exclusion
IC50: Half maximal inhibitory concentration
PD-1 (PDCD1): Programmed cell death protein 1
PD-L1 (CD274): Programmed death-ligand 1
CTLA4: Cytotoxic T-lymphocyte-associated protein 4
LAG3: Lymphocyte-activation gene 3
TIGIT: T-cell immunoreceptor with Ig and ITIM domains
CR/PR: Complete response / Partial response
SD/PD: Stable disease / Progressive disease
TME: Tumor microenvironment
